# Validation of a sleep-disordered breathing screening questionnaire during pregnancy and comparison between mothers and bedpartners prediction of risk

**DOI:** 10.1186/s12884-024-06753-z

**Published:** 2024-08-30

**Authors:** Lauren A. Booker, Mark E. Howard, Susan P. Walker, Danielle L. Wilson

**Affiliations:** 1 Violet Vines Marshman Centre for Rural Health Research, La Trobe Rural Health School, La Trobe University, Edward Street, Bendigo, Victoria Australia; 2 Institute for Breathing and Sleep, Austin Health, Heidelberg, Victoria Australia; 3 Department of Medicine, University of Melbourne, Parkville, Victoria Australia; 4 Department of Obstetrics, Gynaecology and Newborn Health, University of Melbourne, Parkville, Victoria Australia; 5 Mercy Perinatal, Mercy Hospital for Women, Heidelberg, Victoria Australia; 6 School of Electrical Engineering and Computer Science, The University of Queensland, St Lucia, Queensland Australia

**Keywords:** Pregnancy, Obstructive sleep apnea, Polysomnography, Screening, Hypertension

## Abstract

**Background:**

Sleep Disordered Breathing (SDB) in pregnant patients ranges from 3 to 27% and varies depending on gestational age and method used to diagnose. SDB increases the risk of advanced pregnancy complications such as gestational diabetes mellitus, pregnancy-induced hypertension, and preeclampsia. Screening and diagnosis of SDB during pregnancy remains a challenge, with existing screening tools underperforming during pregnancy. This study aimed to validate a previously developed model for predicting SDB during late pregnancy and compare the predictive value of bedpartner responses.

**Methods:**

Ninety-six women in the third trimester of pregnancy underwent polysomnography and completed the Berlin Questionnaire (BQ), with 81 bedpartners completing the BQ about their pregnant partner. A subset of BQ items (snoring volume and tiredness upon awakening) along with BMI > 32 kg/m^2^was utilised to calculate the Wilson Optimized Model (WOM), which demonstrated strong predictive properties in development.

**Results:**

SDB (RDI/hr ≥ 5) was detected in 43.8% of women. BQ identified 72% of pregnant mothers as high risk for SDB (Sensitivity = 83%, Specificity = 37%), compared to 29% of mothers identified by the WOM (Sensitivity = 45%, Specificity = 83%). At RDI of ≥ 15, the WOM correctly classified more women according to SDB risk than the BQ (76.0% vs. 41.7% cases correct,*X*^2^(1) = 23.42,*p* < .001), with no difference at RDI ≥ 5. Bedpartners were more likely to report high risk for SDB on the WOM than pregnant women themselves (38.3% vs. 28.4%), however predictive ability was not improved by bedpartner input (RDI ≥ 5 bedpartner AUC = 0.69 v mother AUC = 0.73).

**Conclusion:**

BQ largely overestimates the prevalence of SDB in pregnancy compared to the WOM which underestimates. Utilising bedpartner responses didn’t improve screening for SDB in late pregnancy. More work is needed to develop a pregnancy-specific tool for quick and accurate screening for SDB.

**Supplementary Information:**

The online version contains supplementary material available at10.1186/s12884-024-06753-z.

## Background

Sleep disordered breathing (SDB) refers to a wide spectrum of sleep-related breathing conditions including snoring, upper airway resistance and regular pauses in breathing [1,2]. Obstructive sleep apnea (OSA) is the most severe form of SDB, and involves cyclical episodes of upper airway collapse causing breathing to stop or reduce during sleep resulting in a fall in blood oxygen levels and sleep disruption [3]. The prevalence of SDB in pregnant patients ranges from 3 to 27% and varies depending on gestational age and method used to diagnose [4,5]. This is substantially higher than nonpregnant reproductive-age females, where the prevalence has been estimated at 0.7 to 6.5% [6,7].

The development or exacerbation of preexisting SDB during pregnancy is complex, with a multitude of physiological changes associated with pregnancy. These changes include weight gain, rhinitis and fluid retention leading to upper airway narrowing and increased airway resistance [8,9], along with progesterone-mediated alternations in ventilation and changes in lung mechanics [10] and the elevation of the diaphragm from the uterus during pregnancy, increasing the risk of SDB for pregnant women [10]. Past research has confirmed that there is a high prevalence of SDB among pregnant populations when screened using polysomnography (PSG; sleep study), particularly in high-risk pregnancies or those with an increased BMI. For example, 37.5–40% of obese cohorts have a respiratory disturbance index (RDI) of 5 or over, indicating SDB [5,11], with SDB occurring in as many as 47% of pregnancies complicated by gestational hypertension or gestational diabetes [4,12].

Recently, there has been a greater understanding of the potential risks associated with undiagnosed SDB during pregnancy including adverse maternal and fetal outcomes, such as pre-eclampsia, gestational diabetes, and impaired fetal growth [13–16]. SDB can increase the risk of advanced pregnancy complications such as gestational diabetes mellitus (GDM), pregnancy-induced hypertension (PIH), and preeclampsia (PEC) [17]. Longitudinal studies have demonstrated that a diagnosis of SDB during pregnancy and postpartum is associated with an increased risk of hypertension and metabolic syndrome up to 7 years after delivery, highlighting the importance of identifying this condition [17,18].

Currently, there are a number of screening questionnaires to help identify individuals with SDB [19]. However, the overall features of these questionnaires, in a non-pregnant population, revolve around advancing age (> 50 years), male sex, and higher body mass index (BMI) that increase SDB risk [20]. For example the Berlin Questionnaire (BQ) [21], the Multivariate Apnoea Prediction Index (MAPI) [22] and the STOP-Bang Questionnaire [23] incorporate age, BMI or gender into the assessment, which favour men and inaccurately measure SDB risk in pregnant woman, as they are usually under the age of 50, not male and have a higher BMI due to weight gain related to their pregnancy. The accuracy of these tools in detecting SDB in pregnant women is therefore poor for this cohort [4,24,25]. The high rates of SDB potentially undetected amongst pregnant women requires urgent focus.

BQ is a common screening questionnaire used in pregnant woman [26–28]. However, it has been shown to be unreliable as a screening tool for SDB in pregnant woman [24]. An optimised predictive model for detecting SDB in pregnancy was developed by Wilson et al. [25], the Wilson Optimized Model (WOM), and has been shown that considering pregnant woman with a BMI of 32 or greater, snoring volume and tiredness upon awakening is more accurate in predicting SDB in pregnant women than the BQ, however this was in a small cohort (*n* = 43).

In addition, snoring and other signs of SDB are hard to self-assess. Most individuals don’t know they are snoring or pausing for breath in their sleep unless they are told by a bedpartner or roommate. Snoring varies considerably and can be classified by its intensity, frequency, and duration. There is a strong association between snoring frequency and intensity and the severity of OSA in non-pregnant populations [29,30], showing the need to be aware when snoring sounds increase in intensity and frequency [31]. Therefore, partner reports might be a better indicator of SDB during pregnancy and be a more accurate predictor than pregnant women themselves. Thus, the aim of the study was to: 1). Validate the WOM against PSG for detecting SDB in pregnancy on a larger sample 2), Compare the WOM against the BQ, which has often been used in pregnancy, and 3). Compare the accuracy of bedpartner responses to the BQ and WOM against the pregnant woman’s scores in predicting SDB risk.

## Method

### Design and participants

Secondary analyses were conducted on a total of 101 pregnant women in their third trimester of pregnancy who were recruited from the antenatal outpatient clinic or pregnancy day assessment centre at the Mercy Hospital for Women, between October 2012 and October 2015, along with 81 of their partners. As part of the study, a diversity of mothers who were diagnosed with hypertension, pre-eclampsia and chronic hypertension [32], as well as BMI- and gestation-matched uncomplicated pregnancies were recruited. The study was approved by the Human Research Ethics Committees at Austin Health (H2012/04469) and Mercy Hospital for Women in Melbourne, Victoria, Australia (R12/02), and all participants gave written informed consent.

### Procedure

Women from 24 weeks’ gestation were approached by a member of the research team and were invited to participate. Once participants consented, a polysomnogram (PSG) was conducted on all participants at their earliest convenience and current BMI was recorded. Within ± 2 weeks of PSG, all participants completed a paper copy version of the Berlin Questionnaire. A copy of this questionnaire, along with an accompanying letter, was voluntarily taken home by the participant for their bedpartner to complete and return.

### Measurement

*Polysomnography (PSG) -*PSG was performed in the Austin Health sleep laboratory using the Compumedics E series (Abbotsford, Victoria, Australia) or if preferred, unattended in the participant’s home using the Somté (Compumedics, Abbotsford, Australia) portable sleep-monitoring device. Sleep and respiratory scoring was done in accordance with current American Academy of Sleep Medicine (AASM) criteria, with the alternative definition used for scoring hypopnoeas [33,34]. The number of apnoeas, hypopnoeas, and respiratory effort related arousals (RERAs) per hour of sleep was calculated and expressed as the respiratory disturbance index (RDI). Thirty-two participants had aPSG in the laboratory and sixty-four had a type-II home sleep study [35].

*Berlin Questionnaire (BQ)-*The Berlin Questionnaire [21] assesses the risk of OSA based on three categories: Category 1, which contains five questions concerning snoring and breathing pauses; Category 2, which containsthree questions on daytime sleepiness; and Category 3, which has a question concerning the history of high blood pressure and a body mass index (BMI) more than 30 kg/m^2^. Scoring of high risk versus low risk of OSA is based on the persistence of these symptoms. As per the questionnaire guidelines, to be considered as high risk for OSA a participant had to qualify as high risk for at least two symptom categories.

*Wilson Optimized Model (WOM)*[25]*-*This model was based on a subset of questions from the Berlin Questionnaire, with the aim to more accurately detect SDB in pregnancy, with a sensitivity of 85%, specificity of 96%, positive predictive value of 92% and negative predictive value of 93% in development. The model comprised of three questions available from the Berlin Questionnaire: (1) Have you been told you snore? (2) How loud is your snoring? and (3) How often do you feel tired after sleeping? along with calculation of BMI > 32 kg/m^2^(see Appendix TableA1). A score was allocated to the response to each question, with higher scores relating to higher risk of SDB, with a possible total score ranging between 2.2 and 25.2. If the total score was more than 18.1, the participant was considered high risk of SDB.

### Statistical analysis

Statistical analyses were performed using SPSS 21 (SPSS Inc., Chicago, Illinois). SDB was defined as an RDI ≥ 5 events per hour, with secondary analyses performed at an RDI ≥ 15 events per hour as per PSG results. A two-sided*p*-value < 0.05 was considered statistically significant. Using the Wilson Optimized Model criteria, the new scores were tallied to provide for an overall score, with > 18.1 considered high risk for SDB. Data was then recoded into a categorical variable (Yes/No). Cross-tabulations were performed to determine sensitivity and specificity values for the Wilson and Berlin Questionnaires as completed by the pregnant participant and bedpartner. Between group comparisons were conducted using chi-square analysis to determine the accuracy of the Berlin Questionnaire and Wilson Optimized Model for the pregnant participant, and the Wilson Optimized Model for the pregnant participant versus the bedpartner against PSG scores in predicting SDB. Receiver operator characteristic (ROC) curves were generated and the areas under the curves (AUC) calculated to assess the diagnostic performance of the Wilson Optimized Model against RDI scores for both pregnant participants and their partners. ROC curves could not be performed for the Berlin Questionnaire as it is a binary (high vs. low risk) predictor.

## Results

Of the 101 pregnant mothers recruited for the study, 96 completed the Berlin Questionnaire (Mean ± SD: age = 33.3 ± 4.5 years, gestational age at time of sleep study = 33.2 ± 3.2 weeks, BMI at time of sleep study = 35.8 ± 6.3 kg/m^2^). Fifty-two (54%) had a hypertensive disorder of pregnancy (16 with preeclampsia, 22 with gestational hypertension, and 14 with chronic hypertension). The median (IQR) RDI/hr was 4.3 (2.2, 9.3) with mild SDB (RDI ≥ 5) found in 42 of the 95 women (43.8%), and moderate-severe SDB (RDI ≥ 15) in 15 (15.6%) women. All reported a stable bed partner for the pregnancy and 53.1% were nulliparous.

### Berlin versus Wilson Optimized Model

For the Berlin Questionnaire, 69 (71.9%) pregnant mothers were at high risk of SDB, whereas less were at risk when using the Wilson Optimized Model with 28 (29.2%) at high risk of SDB (Supporting TableS1). Table 1gives the diagnostic test thresholds for the Berlin and Wilson Optimized Models. Whilst the Berlin Questionnaire was more sensitive (83.3%) than the Wilson Optimized Model (45.2%) at picking up those who have mild SDB, there was a high number of false positives with low specificity (37.0%, compared to the Wilson Optimized Model = 83.3%). A similar pattern of results was seen for moderate-to-severe SDB (RDI ≥ 15) with a trade-off between higher sensitivity for the Berlin Questionnaire (93%) compared to higher specificity for the Wilson optimized model (78%; Table 1). A comparison between the two questionnaires showed that at a cut-off of RDI ≥ 5, the Berlin Questionnaire was able to classify 57.3% of the cases correctly according to risk of SDB compared to 66.7% for the Wilson Optimized Model (*X*^2^(1) = 1.79,*p* = .23). However, at an RDI ≥ 15, the Wilson Optimized Model was significantly more accurate than the Berlin Questionnaire at classifying cases according to SDB risk (76.0% of cases correct vs. 41.7%,*X*^2^(1) = 23.42,*p* < .001).

**Table 1:** Diagnostic comparison between the Berlin and Wilson optimized model for the pregnant mother and bedpartner for RDI ≥ 5 & ≥ 15

	Mother – Berlin		Mother – Wilson		Partner – Wilson	
	RDI ≥ 5	RDI ≥ 15	RDI ≥ 5	RDI ≥ 15	RDI ≥ 5	RDI ≥ 15
Sensitivity (%)	83.3	93.3	45.2	66.7	51.3	64.3
Specificity (%)	37.0	32.1	83.3	77.8	73.8	67.2
Likelihood ratio (relative risk)	1.32	1.37	2.71	3.00	1.96	1.96
PPV (%)	50.7	20.3	67.9	35.7	64.5	29.0
NPV (%)	74.1	96.3	66.2	92.6	62.0	90.0

*Note*RDI, Respiratory Disturbance Index; PPV, positive predictive value; NPV, negative predictive value

### Pregnant mother versus bedpartner questionnaire

The Berlin Questionnaire was completed by 81 of the bedpartners, and hence 81 scores on the Wilson Optimized Model were calculated and compared to the pregnant mother. In this group, bedpartners were equally likely to report the pregnant women to be at high risk of SDB based on the Berlin Questionnaire (*n* = 59 out of 81, 72.8%; TableS2), however they were more likely to report high risk of SDB based on the Wilson Optimized Model (*n* = 31 out of 81, 38.3%) compared to the mother’s own reports (*n* = 23 out of 81, 28.4%,*p* = .24).

Agreement between the mother and bedpartner on Wilson Optimized Model risk scores was 75.3% (Table 2), with discrepancies more likely to occur when the bedpartner reported high risk of SDB against the mother’s low risk score (17.3%). Of the 23 mothers who either reported that they did snore at all (*n* = 13) or did not know if they snored (*n* = 10), 12 bedpartners said that they did. In addition, bedpartners were more likely to report the pregnant mother as being tired every day (*n* = 31) compared to the mother (*n* = 24).

**Table 2:** Comparison of mother against bedpartner scores for Wilson Optimized Model

		Partner		
**Mother**		High Risk	Low Risk	Total
	High Risk	17 (21.0%)	6 (7.4%)	23
	Low Risk	14 (17.3%)	44 (54.3%)	58
	Total	31	50	81

The discriminatory ability of the Wilson Optimized Model to predict SDB at an RDI ≥ 5 when completed by the bedpartner was more sensitive than when completed by the mothers, but less specific (Table 1). The ability of the Wilson Optimized Model to accurately classify cases of either mild or moderate SDB was not improved by the bedpartners input above what can be provided by the mother (RDI ≥ 5 – mother = 66.7% vs. bedpartner = 63.0%,*X*^2^(1) = 0.26,*p* = .64; RDI ≥ 15 – mother = 76.0% vs. bedpartner = 66.7%,*X*^2^(1) = 1.91,*p* = .18).

**Fig. 1:**
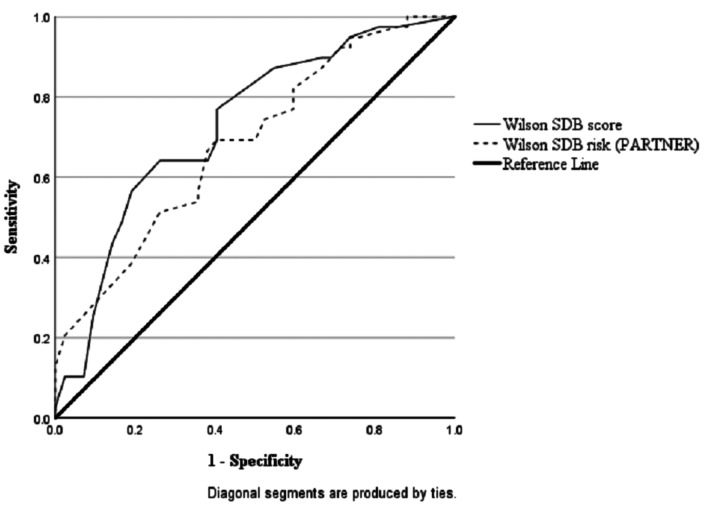
ROC curve analysis between mothers’s Wilson scores (AUC = .73) and partner’s Wilson scores (AUC = .69) against PSG RDI scores = 5

**Fig. 2:**
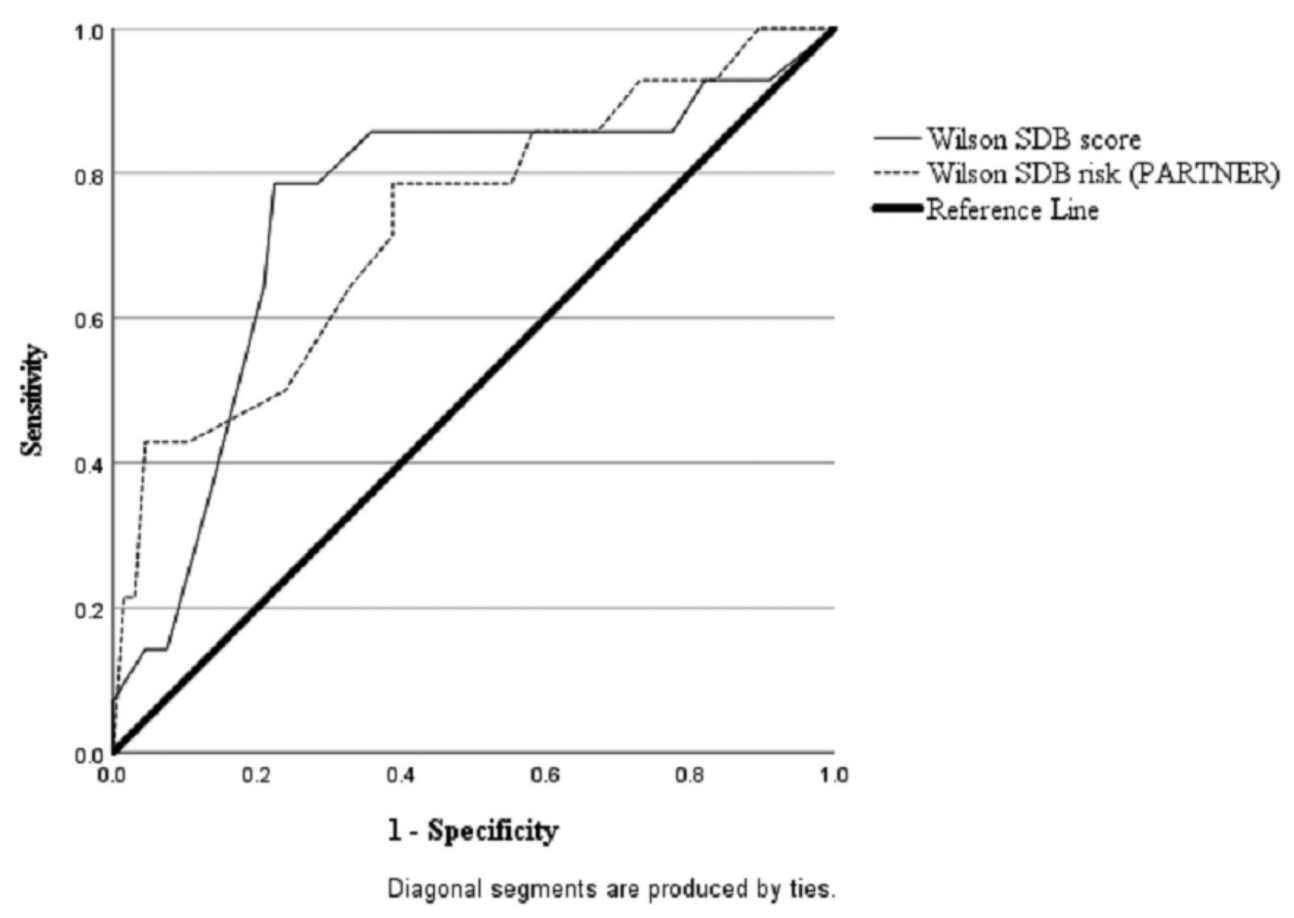
ROC curve analysis between mothers’s Wilson scores (AUC = .73) and partner’s Wilson scores (AUC = .69) against PSG RDI scores = 5

The ROC curves show that the predictive accuracy of the Wilson Optimized Model completed by the mother compared to PSG scores of RDI ≥ 5 was good with an area under the curve (AUC) of 0.73 (C.I.= 0.62-0.84,*p* = < 0.001; Fig. 1). The model based on the bedpartner scores was also significant, but the AUC was poorer (AUC = 0.69, C.I = 0.57-0.80,*p* = .002; Fig. 1). However, the diagnostic performance of the mother’s responses was not significantly better than the bedpartner’s (*X*^2^(1) = 1.04,*p* = .31). The results for both mother and bedpartners scores on the Wilson Optimized Model were fair in predicting RDI ≥ 15 (mother AUC = 0.75, C.I = 0.59 − 0.90,*p* = .002; vs. bedpartner AUC = 0.73, C.I = 0.58–89,*p* = .003; Fig. 2). In this sample, 52 out of 95 mothers (54.7%) had a hypertensive disorder of pregnancy (HDP). Sub-analysis within this group showed that the ability of the screening tools to classify cases of SDB (RDI ≥ 5) correctly according to PSG was not different for women with a HDP compared to normotensive pregnant women (Berlin Q – HDP = 63.5% vs. normotensive = 50.0%,*X*^2^(1) = 1.77,*p* = .22; Wilson model – HDP = 73.1% vs. normotensive = 59.1%,*X*^2^(1) = 2.10,*p* = .19). However, the Wilson Optimized Model did show promising utility as a ‘rule-in’ tool, with a PPV of 83.3% meaning that 83% of hypertensive women screening positive would be given a diagnosis of SDB on PSG (sensitivity = 57.7%, specificity = 88.5%, LR = 5.0, NPV = 67.6%).

## Discussion

We confirm that the Wilson Optimized Model was more accurate than the Berlin Questionnaire at discriminating cases of moderate-to-severe SDB diagnosed on PSG during pregnancy, with better specificity, a higher PPV and a comparable NPV. However, the screening tools had contrasting properties with similar success rates for mild SDB at an RDI ≥ 5. The Berlin Questionnaire largely overestimated the prevalence of SDB with only half of those with a high-risk Berlin Questionnaire score having SDB. In contrast, the Wilson Optimized Model underestimated the prevalence of OSA in pregnant mothers, with a third of women at low risk for SDB demonstrating objectively measured mild SDB. It could be argued that missing a diagnosis of mild OSA is of less clinical significance, however. Furthermore, utilising bedpartner responses to the mothers’ SDB symptoms did not improve screening for SDB in late pregnancy.

The first aim of this study was to validate the Wilson Optimized Model. The findings from these data are different to the original paper published (Wilson et al., 2013), with a drop in sensitivity from 85 to 45% and a drop in specificity from 96 to 83%. This may be due to several reasons, including the higher prevalence of hypertensive disorders in this cohort, and differences in gestational age across studies, as the utility of screening tools varies at different stages of pregnancy. However, BMI and prevalence of SDB was equivalent across cohorts, so the degree of discrepancy in predictive properties across cohorts is unexpected.

SDB has been a predominately male disease, and as such screening tools have been developed with male characteristics. However, woman with SDB are more likely to have comorbidities of diabetes, hypertension, cardiac disease and depression, compared to men [36–38]. Furthermore, the phenotype of SDB in women has been described as distinct from men. The clinical presentation of SDB also differs, with men more likely to complain of typical symptoms such as snoring and witnessed apneas, whereas women present with more nonspecific complaints such as fatigue, tiredness, sleep onset insomnia, and morning headaches [39]. Current screening tools are geared towards male symptomatology, and consequently the inclusion of more females in cohorts screening for SDB results in less diagnostic accuracy of SDB screening tools [40]. As women are less likely to present with classic symptoms of SDB, there is a need to develop a screening tool specifically to accommodate women. Additionally, screening for SDB in a pregnant population is also important to identify, due to additional risks such a high blood pressure, pregnancy-induced hypertension, gestational diabetes mellitus (GDM), pre-eclampsia, slowed fetal growth and pre-term birth [15,41–43]. During pregnancy, changes such as weight gain may not impact on SDB risk in the same way as obesity outside of pregnancy, with hormonal, physiological and physical changes also likely to contribute to increased risk [10]. In addition, pregnant women often feel tired and fatigued for reasons outside of apnea-related sleep disruption [44], resulting in traditional screening tools being potentially inaccurate. Overall, the Wilson Optimized Model had the ability to better predict cases of higher severity SDB in pregnancy with an RDI ≥ 15 compared to the Berlin Questionnaire, but both tools were not overly discriminatory for mild SDB requiring further research to be conducted in this area.

The Wilson Optimized Model attempted to account for some of the differences in SDB risk related to pregnancy by using a modified version of the Berlin Questionnaire developed in a pregnant cohort. However, at the previously recommended cut-off for high risk for SDB there was a high number of false negatives, suggesting that mothers at risk of OSA are being missed with this screening tool. The Wilson Optimized Model may show better utility than the Berlin Questionnaire as a “rule-in” tool with two-thirds of those scoring high risk being diagnosed with at least mild SDB (and 83% of hypertensive women), and an improved rate of picking up clinically significant severe disease, but its predictive properties are unlikely robust enough to introduce into clinical practice at this stage. As this study focused on validating a previously developed screening tool, we did not widen the search for potential predictors of SDB. Others have found similar combinations to ours that successfully screen for SDB in their cohorts, such as age, BMI and frequent snoring, with [28] or without chronic hypertension [45], whereas others have introduced anthropometric predictors such as Mallampati class [46] and tongue enlargement [47]. Differences in results are likely due to the trimester of screening and method of SDB diagnostic device, high risk vs. low risk cohorts, and high heterogeneity of participant demographics.

The key strength of this study was the addition of an SDB screening questionnaire specifically addressed to the participant’s bedpartner. This study was novel in the use of questionnaires specifically targeted to the bedpartner, where the Wilson Optimized Model demonstrated a 25% disagreement in risk between mothers and their bedpartners. This was more often due to the bedpartner rating the risk of SDB higher than the mother, due to increased reports of snoring and daily tiredness. Bedpartners may be more accurate in screening for SDB due to being able to judge the presence, and volume, of partner snoring [29,30]. Alternately, snoring may be under reported in women because they are less likely to be accompanied by their bedpartner during clinical evaluation [48]. This study, however, found that bedpartners were not more accurate than the pregnant women in predicting their OSA risk. Only one other study included bedpartner reports of sleep apnoea symptomatology [47]. They found that adding bedpartner information to the combination of the Sleep Apnea Symptom Score, age and BMI demonstrated better predictive value in detecting OSA, although predictive values and AUC only marginally improved. The bedpartner information gathered in the Izci Balserak et al. study was via the pregnant woman asking for this information, whereas we specifically asked the bedpartner to complete the questionnaire and return it in a sealed envelope, removing potential bias. This study also used full polysomnography to characterise SDB, enabling detection of milder respiratory events and those associated with arousals from sleep, in contrast to other studies using limited channel devices [28,45,49]. Previous studies have shown that there is a higher prevalence of SDB in mothers with hypertension and obesity [50,51]. A limitation of this study is that the sample consisted of both hypertensive and BMI-matched normotensive pregnant women, therefore there is likely a higher prevalence of SDB in this study and our results may not be readily transferable to a low-risk population. Alternatively, our cohort represents those at highest risk of SDB during pregnancy who would benefit the most from SDB screening.

## Conclusion

This study highlights the need for an accurate screening tool for SDB in pregnant woman. Existing questionnaires are based on gender and male-associated symptoms, and may lack accuracy in this population. Although performing better than the Berlin Questionnaire, within this cohort the Wilson Optimised Model only demonstrated fair predictive capacity. This study is unique as it compared the accuracy of SDB screening from both the perspective of the pregnant individual as well as the bedpartner. However, insights from the bedpartner did not improve prediction of SDB during pregnancy.

## Electronic supplementary material

Below is the link to the electronic supplementary material.

Supplementary Material 1

## Data Availability

The data that support the findings of this study are available from the corresponding author upon reasonable request.

## Abbreviations

BMI: Body Mass Index
GDM: Gestational Diabetes Mellitus
OSA: Obstructive Sleep Apnoea
PSG: Polysomnography
PPV: Positive Predictive Value
NPV: Negative Predictive Value
RDI: Respiratory Disturbance Index
SDB: Sleep Disordered Breathing
