# Correction: Validation of a sleep-disordered breathing screening questionnaire during pregnancy and comparison between mothers and bedpartners prediction of risk

**DOI:** 10.1186/s12884-024-06808-1

**Published:** 2024-09-17

**Authors:** Lauren A. Booker, Mark E. Howard, Susan P. Walker, Danielle L. Wilson

**Affiliations:** 1 Violet Vines Marshman Centre for Rural Health Research, La Trobe Rural Health School, La Trobe University, Edward Street, Bendigo, VIC Australia; 2 Institute for Breathing and Sleep, Austin Health, Heidelberg, VIC Australia; 3 Department of Medicine, University of Melbourne, Parkville, VIC Australia; 4 Department of Obstetrics, Gynaecology and Newborn Health, University of Melbourne, Parkville, VIC Australia; 5 Mercy Perinatal, Mercy Hospital for Women, Heidelberg, VIC Australia; 6 School of Electrical Engineering and Computer Science, The University of Queensland, St Lucia, QLD Australia

**Correction: BMC Pregnancy Childbirth 24, 565 (2024)**

10.1186/s12884-024-06753-z

Following publication of the original article [1], the authors reported an error in Author’s affiliations. Affiliation 6 should be affiliated to author Danielle L. Wilson and not with Lauren A. Booker.

The author group has been updated above.

The author also reported some error in the text. Changes are marked as bold.

- The first line of the abstract should read “Sleep **Disordered** Breathing” rather than **Disorder**.
- Heading on page 3: the **Wilson Optimized Model** for the heading “Berlin versus **wilson optimized model**” should be capitalised.
- Under Abbreviations on page 7, “**GDM7**” should be “**GDM**”.

The original article [1] has been corrected.

## References

[1] Booker LA, Howard ME, Walker SP, et al. Validation of a sleep-disordered breathing screening questionnaire during pregnancy and comparison between mothers and bedpartners prediction of risk. BMC Pregnancy Childbirth. 2024;24:565. 10.1186/s12884-024-06753-z. 39215252 10.1186/s12884-024-06753-z PMC11363667

