# Supplementary material for: Validation of a sleep-disordered breathing screening questionnaire during pregnancy and comparison between mothers and bedpartners prediction of risk
Source: BMC Pregnancy Childbirth. 2024 Aug 30;24:565. doi: 10.1186/s12884-024-06753-z (PMC11363667; doi:10.1186/s12884-024-06753-z)
Supplement: Supplementary file 1 — Supplementary Material 1 [file 12884_2024_6753_MOESM1_ESM.docx]

# *Table A1. Wilson Optimized Model for SDB screening during pregnancy*

# **Wilson Optimized Model**

| **Risk Factor** | **Scoring (circle as appropriate)** |
| --- | --- |
| **1. Does the patient snore? Berlin Q1** | Yes = Go to Q2  No or Don’t Know = Go to Q3 |
| **2. How loud is the snoring? Berlin Q2** | 1. as loud as breathing Score = 2.4  2. as loud as talking Score = 4.8  3. louder than talking Score = 7.2  4. very loud Score = 9.6  5. no snoring Score= 0  6. Don’t know Score = 0 |
| **3. How often does the patient feel tired**  **after sleeping? Berlin Q6** | 1. Never or nearly never Score = 2.2  2. 1-2 times a month Score = 4.4  3. 1-2 times a week Score = 6.6  4. 3-4 times a week Score = 8.8  5. Nearly every day Score = 11  6. Don’t know Score = 0 |
| **4. Is the patient’s current BMI greater**  **than 32kg/m^2^?** | Yes No  Score = 4.6 Score = 0 |

**Total Score = _______(Q2) + _______(Q3) + _______(Q4) = __________**

Score range should be from 2.2 to 25.2. If total score is more than 18.1, the patient is at very high risk of SDB.

*Supporting Tables*

*Table S1. Chi square analyses comparing mother’s Berlin and Wilson scores against RDI ≥ 5 and RDI ≥ 15.*

|  | **RDI ≥ 5** | |  |  |
| --- | --- | --- | --- | --- |
| **Berlin** | **Yes** | **No** | **Total** | ***P*** |
| High risk | 35 (50.7%) | 34 (49.3%) | 69 (100%) | .04 |
| Low risk | 7 (25.9%) | 20 (74.1%) | 27 (100%) |  |
| **Wilson** | **Yes** | **No** | **Total** | ***P*** |
| High risk | 19 (67.9%) | 9 (32.1%) | 28 (100%) | .003 |
| Low risk | 23 (33.8%) | 45 (66.2%) | 68 (100%) |  |
| **RDI ≥ 15** | | | |  |
| **Berlin** | **Yes** | **No** | **Total** | ***P*** |
| High risk | 14 (20.3%) | 55 (79.7%) | 69 (100%) | .06 |
| Low risk | 1 (3.7%) | 26 (96.3%) | 27 (100%) |  |
| **Wilson** | **Yes** | **No** | **Total** | ***P*** |
| High risk | 10 (35.7%) | 18 (64.3%) | 28 (100%) | .001 |
| Low risk | 5 (7.4%) | 63 (92.7)% | 68 (100%) |  |

n = 96

*Table S2. Chi square analyses comparing bedpartner’s Berlin and Wilson scores against RDI ≥ 5 and RDI ≥ 15.*

|  | **RDI ≥ 5** | |  |  |
| --- | --- | --- | --- | --- |
| **Berlin** | **Yes** | **No** | **Total** | ***P*** |
| High risk | 31 (52.5%) | 28 (47.5%) | 59 (100%) | .22 |
| Low risk | 8 (36.4%) | 14 (63.6%) | 22 (100%) |  |
| **Wilson** | **Yes** | **No** | **Total** | ***P*** |
| High risk | 20 (64.5%) | 11 (35.5%) | 31 (100%) | .02 |
| Low risk | 19 (38.0%) | 31 (62.0%) | 50 (100%) |  |
| **RDI ≥ 15** | | | |  |
| **Berlin** | **Yes** | **No** | **Total** | ***P*** |
| High risk | 13 (22.0%) | 46 (78.0%) | 59 (100%) | .10 |
| Low risk | 1 (4.6%) | 21 (95.5%) | 22 (100%) |  |
| **Wilson** | **Yes** | **No** | **Total** | ***P*** |
| High risk | 9 (29.0%) | 22 (71.0%) | 31 (100%) | .04 |
| Low risk | 5 (10.0%) | 45 (90.0)% | 68 (100%) |  |

n = 81
